# Protein Oxidation and Sensory Quality of Brine-Injected Pork Loins Added Ascorbate or Extracts of Green Tea or Maté during Chill-Storage in High-Oxygen Modified Atmosphere

**DOI:** 10.3390/medicines5010007

**Published:** 2018-01-15

**Authors:** Sisse Jongberg, Mari Ann Tørngren, Leif H. Skibsted

**Affiliations:** 1 Department of Food Science, University of Copenhagen, Rolighedsvej 30, DK-1958 Frederiksberg, Denmark; ls@food.ku.dk; 2 Danish Meat Research Institute, Danish Technological Institute, Gregersensvej 9, DK-2630 Taastrup, Denmark; matn@teknologisk.dk

**Keywords:** brine-injected pork, green tea extract, maté extract, ascorbate, protein oxidation, sensory quality, high-oxygen modified atmosphere packaging

## Abstract

**Background:** Ascorbate is often applied to enhance stability and robustness of brine-injected pork chops sold for retail, but may affect protein oxidation, while plant extracts are potential substitutes. **Methods:** Brine-injected pork chops (weight-gain ~12%, NaCl ~0.9%) prepared with ascorbate (225 ppm), green tea extract (25 ppm gallic acid equivalents (GAE)), or maté extract (25 ppm GAE) stored (5 °C, seven days) in high-oxygen atmosphere packaging (MAP: 80% O_2_ and 20% CO_2_) were analyzed for color changes, sensory quality, and protein oxidation compared to a control without antioxidant. **Results:** No significant differences were observed for green tea and maté extracts as compared to ascorbate when evaluated based on lipid oxidation derived off-flavors, except for stale flavor, which maté significantly reduced. All treatments increased the level of the protein oxidation product, α-aminoadipic semialdehyde as compared to the control, and ascorbate was further found to increase thiol loss and protein cross-linking, with a concomitant decrease in the sensory perceived tenderness. **Conclusions:** Green tea and maté were found to equally protect against lipid oxidation derived off-flavors, and maté showed less prooxidative activity towards proteins as compared to ascorbate, resulting in more tender meat. Maté is a valuable substitute for ascorbate in brine-injected pork chops.

## 1. Introduction

Meat produced and distributed for the food service sector, canteens, nursing homes, and schools needs more robustness to withstand common practice, which includes several cycles of heating often to high temperatures (usual above 75 °C) and subsequent chilling [1,2]. Addition of salt binds water in the meat, and results in a weight-gain and improved tenderness and juiciness, which collectively gives a more robust meat product that better tolerates several heating/chilling cycles [3]. Meat cuts sold for the food service sector are distributed both in modified atmosphere packaging (MAP) and in vacuum, and are stored chilled or frozen (Nassu, Juarez, Uttaro, & Aalhus, 2010). As reviewed by Suman et al. [4], high-oxygen MAP improves meat color, but impairs the eating quality by accelerating oxidation of lipids and proteins, resulting in off-flavor formation, and decreased tenderness and reduced juiciness. High-oxygen MAP has, moreover, been found to reduce the otherwise positive effects of injection-enhancement on shear force, tenderness, and juiciness of beef steaks and concomitantly increase off-flavor associated with lipid oxidation [3].

Ascorbate is commonly added to injection brines as an antioxidant for protecting color and lipids of fresh and frozen meat products, as it ensures a stable and robust meat product [5]. However, there is a risk that ascorbate may act as a prooxidant at some concentration levels depending on the system [6]. Tea catechins have previously shown superior antioxidant activity when compared to ascorbate during cold storage of cooked or raw beef or chicken patties [7]. Hence, plant extracts may be potential “natural” substitutes for ascorbate to extend the shelf life of brine-injected meat. Such natural antioxidants extracted from plant material rich in phenolic compounds often protect efficiently against lipid oxidation in meat when added to the animal feed [8,9], when mixed into minced meat products [7,10], or when added to brine for whole meat cuts [11,12]. However, most studies considering the effects of natural antioxidants in meat have explored the effects in minced meat or in surface-marinated meat products. Brine-injection is commonly applied to improve tenderness and juiciness; however, the effects of phenolic antioxidants in brine-injected meat products have not been widely researched. Protein oxidation is known to decrease meat tenderness and juiciness in fresh meat [13], and the effects of phenolic antioxidants in the brine on protein oxidation and meat tenderness need accordingly to be considered. Green tea (*Camellia sinensis*) is commonly used as an antioxidant in various food products [14], whereas maté (*Ilex paraguariensis*) from a South American bush rich in caffeic acid derivatives [15] is a new and less utilized source of phenolic antioxidants for foods. Recently, it was shown that maté extract added to cattle feed resulted in more tender meat with higher consumer acceptance [16]. Furthermore, studies show that extracts from green tea and maté injected into pork loins protected against lipid oxidation during chilled storage in a dose-dependent manner, but found that the meat protein radical intensity increased when added increasing doses of green tea extract [17]. This indicated a prooxidative effect of green tea extract on the meat proteins, which needs to be investigated further.

The aim of the present study was to investigate the effect of substituting ascorbate as an antioxidant agent in brine-injected pork loins with phenolic-rich extracts from green tea or maté. Effects on meat protein oxidation as evaluated by thiol loss and formation of protein disulfide cross-link during storage in high-oxygen atmosphere were related to product color and sensory quality including tenderness and juiciness.

## 2. Materials and Methods

### 2.1. Plant Extracts and Chemicals

Green tea (*Camellia sinensis*) extract (Guardian Green Tea 20 M), a commercial product with Product description PD 215033-6.0EN) was obtained from DuPont Nutrition and Biosciences ApS, Brabrand, Denmark. Maté (*Ilex paraguariensis*) extract from Centroflora, Botucatu, Sao Paulo, Brazil, was kindly provided by Daniel Cardoso at University of Sao Paulo in Sao Carlos. Details regarding extract composition and extraction method are previously published by de Zawadzki et al. [16]. All other reagents were of analytical grade. Double-deionized water (Milipore, Bedford, MA, USA) was used throughout.

### 2.2. Total Phenolic Content in Extracts

In order to control the amount of phenols injected into the pork, the total phenolic content was determined in the maté extract by Folin–Ciocalteu’s method as described by Singleton and Rossi [18]. The total phenolic content in the green tea extract was previously determined to be 23.8% [19], and this concentration was applied throughout this investigation. In brief, 100 µL 1 mg/mL maté extract dissolved into a total volume of 1500 µL double-deionized water, was left to react with 125 µL Folin–Ciocalteu phenol reagent (Sigma-Aldrich, St. Louis, MO, USA) for 8 min. Subsequently, 375 µL 20% sodium carbonate was added and the reaction mixture was left to incubate at 20 °C for 2 h. The phenol concentration was determined spectrophotometrically at 765 nm against a standard curve prepared from gallic acid. The concentrations are given in gallic acid equivalents (g GAE/100 g dry extract; % *w*/*w*).

### 2.3. Preparation and Storage of Injected Pork Loins

Thirty-six pork loins (*logissimus dorsi*) from 18 female pigs slaughtered on the same day and with similar and normal pH (5.61 ± 0.06) were collected from a Danish slaughterhouse and transported to the Danish Meat Research Institute at Technological Institute (Taastrup, Denmark). Pigs 1–6 were used for day 0 samples, pigs 7–12 were used for day 3 samples, and pigs 13–18 were used for day 7 samples under the assumption that six replicates would compensate for any animal variation between the 18 pigs. The loins were divided in half, and hip-ends and neck-ends from the same pig were randomized and injected with either a salt brine, or salt brine with ascorbate, green tea or maté extract, resulting in six replicates (A–F) for each treatment at each day of sampling. The salt brine contained 6.6% NaCl and 5.5% dextrose. The brine containing ascorbate was added 0.21% sodium ascorbate, corresponding to ~225 ppm in the injected loins. The brine containing green tea was added 0.10% green tea extract, and the brine containing maté was added 0.11% maté extract, resulting in a similar level of ~25 ppm phenolic compound in the injected loins with an expected weight gain of 12% (*w*/*w*). The half-loins were weighted prior to injection to determine the exact gain after injection, which were performed using a multichannel brine injector (FMG 26/52, Fomaco A/S, Køge, Denmark) with 66 punch/min, 1.0 bar pressure, and 3.0 bar up-pressure for the hip ends and 66 punch/min, 0.8 bar pressure, and 3.0 bar up-pressure for the neck end. The injected half-loins were covered in plastic bags to avoid evaporation from the surface and left to equalize overnight in the dark at 2–5 °C. The day after brine-injection, the loins were blotted from drip-loss and weighed for calculation of weight-gain. The weight-gain was further used to calculate the exact amount of ascorbate and phenolic compounds present in the meat:

Calculation example:

(1) Phenol content in brine: 0.10% extract in brine gives 0.1 g/100 mL·23.8% = 0.238 mg/mL phenol in brine;
(2) Phenol in loin (2.02 kg) when weight-gain was 193 g (mL): 193 mL·0.238 mg/mL phenol = 44.4 mg phenol;
(3) Phenol (ppm) in loin: 44.4 mg phenol/2.02 kg meat = 22.3 mg phenol/kg meat = 22.3 ppm.

The half-loins were subsequently sliced into 8 chops of 2 cm and numbered 1–16, having numbers 1–8 starting from the hip-end and 9–16 ending in the neck-end. The pork chops were randomized from the various analyses, and the pork chops to be analyzed on day 0 were vacuum packed and frozen (−80 °C) until analysis. The pork chops to be stored for day 3 and day 7 were packed (two in each tray) in modified atmosphere packaging (MAP, 80% O_2_/20% CO_2_) using a Multivac T200 tray sealer (Multivac, Wolfertschwenden, Germany). Tray (MAPET K 2190-53H, clear) and film (Toplex HB PET EP 40 code 2600/040) with oxygen permeability: 2.5 cc/m^2^/24 h were obtained from Færch Plast (Holstebro, Denmark). The MAP pork chops were stored for 3 or 7 days at 5 °C in a display cabinet with light exposure (~1200 lux as measured on product surface) for 12 h daily. After sampling, the surface color was measured before mincing and mixing. The meat was divided into smaller portions, vacuum packed, and stored at −80 °C until analysis.

### 2.4. Salt Analysis

The concentration of salt was determined according to Nordisk Metodikkomite for Levnedsmidler [20]. In brief, sodium chloride was extracted in hot water, and chloride was subsequently precipitated using silver nitrate. The concentration of salt was determined in 72 samples (from 18 pigs injected with four different brines) and was calculated as an average of a pork chop from the middle of the loin and from the end. Results are presented in g/100 g as mean ± sd (*n* = 6).

### 2.5. Color Analysis

The surface color of the pork chops was measured by a videometer (VideometerLab, Videometer A/S—Visionteknologi, Hørsholm, Denmark). Spectra consisting of 1.2 mil pixel were obtained from which lightness (L*), and color (a* and b*) were calculated from the ratio between specific reflection values in correspondence with the original CIE definitions. On each day of take-out (day 0, 3 and 7), 24 samples (4 brines × 6 replicates) were measured. From L*, a* and b* the total color difference (ΔE) was calculated [21]:

ΔE = √ (ΔL^2^ + Δa^2^ + Δb^2^).

### 2.6. Isolation of Myofibrillar Proteins (MPI)

Myofibrillar proteins (MPI) were isolated from the pork loins according to the method by Park, Xiong, and Alderton [22] with slight modifications [23]. From each combination of injection brine and day, six replicates were prepared (*n* = 6). The MPI were lyophilized and stored at −20 °C until analysis.

### 2.7. Protein Thiol Concentration

Protein thiol groups were determined in the MPI after derivatization with DTNB (5,5 dithiobis(2-nitrobenzoic acid, Sigma-Aldrich, St. Louis, MO, USA)) according to Ellman [24] as previously described in detail [25].

### 2.8. SDS-PAGE

MPI were analyzed by gel-electrophoresis using NuPAGE Novex 3–8% TRIS-acetate gels according to the manufacturer’s instructions (Invitrogen, Carlsbad, CA, USA) and as described by Jongberg et al. [25]. Precision Plus Protein Standard All Blue marker was used as protein marker and loading control on the gels. The gels were photographed by a charge-coupled device (CCD) camera (Raytest, Camilla II, Straubenhardt, Germany) and the protein bands were quantified using GelAnalyzer 2010^©^ developed by Dr. Istvan Lazar.

### 2.9. Determination of α-Aminoadipic Semialdehyde (AAS)

A standard of *N*α-acetyl-α-aminoadipic semialdehyde (AAS) was synthesized from *N*α-acetyl-l-lysine using lysyl oxidase activity from egg shell membrane following the procedure described by Akagawa et al. [26] with slight modifications. Briefly, 10 mM *N*α-acetyl-l-lysine was incubated with constant stirring with 3 g egg shell membrane in 50 mL of 20 mM sodium phosphate buffer, pH 9.0 at 37 °C for 24 h. The egg shell membrane was then removed by centrifugation and the pH of the solution adjusted to 6.0 using 1 M HCl. The resulting aldehyde were reductively aminated with 3 mmol p-amino-benzoic acid (ABA) in the presence of 4.5 mmol sodium cyanoborohydride NaBH3CN at 37 °C for 2 h with constant stirring. Then, the AAS-ABA derivative was hydrolyzed by 50 mL of 12 M HCl at 110 °C for 18 h. The hydrolysates were adjusted to neutral pH 7 using 2.0 M and 0.5 M NaOH and dried in vacuo (40 °C) over night. The purity of the resulting solution and authenticity of the standard compounds obtained following the aforementioned procedures have been checked by using MS and ^1^H NMR [27,28].

MPI was derivatized with ABA according to the procedure by Utrera et al. [29] with slight modifications. An aliquot of 5.0 mg MPI was treated with 0.5 mL 1% sodium dodecyl sulfate (SDS) and 1 mM diethylenetriaminepentaacetic acid (DTPA), 0.5 mL of 50 mM ABA, and 0.25 mL of 100 mM NaBH3CN. All solutions prepared in 250 mM 2-(*N*-morpholino) ethanesulfonic acid (MES) buffer pH 6.0 and were freshly made at the day of analysis. The derivatization was completed by allowing the mixture to react for 90 min while tubes were immersed in a water bath at 37 °C and stirred every 30 min. The derivatization reaction was stopped by adding 0.25 mL of cold 50% tricholoacetic acid (TCA) followed by a centrifugation at 5000 rpm (4 °C) for 5 min, and subsequently the supernatant was discarded. The proteins were further purified by precipitation with 1.5 mL ice cold 5% TCA followed by a centrifugation at 5000 rpm (4 °C) for 5 min, and subsequently the supernatant was discarded. Protein hydrolysis was performed at 110 °C for 18 h after addition of 1.0 mL 6 M HCl. The hydrolysates were adjusted to neutral pH 7 using 2.0 M and 0.5 M NaOH and dried in vacuo (40 °C) over night. Hydrolysates were finally reconstituted with 1.0 mL Milli-Q water and filtered through hydrophilic polypropylene syringe filters (0.22 μm pore size) for HPLC analysis.

AAS in the ABA-derivatized MPI was quantified using ultrahigh-pressure liquid chromatography (UHPLC, Dionex Ultimate 3000, Thermo Fischer Scientific, Hvidovre, Denmark) equipped with an Accucore-150-C18 column (10 cm × 2.1 mm × 2.6 μm) with guard (Def pk4) (Thermo Fischer Scientific, Hâgersten, Sweden) coupled to a fluoresecnece detector (FLD, Dionex Ultimate 3000, Thermo Fischer Scientific, Hvidovre, Denmark) with excitation wavelength of 283 nm and emissions wavelength of 350 nm. The mobile phase was a mixture of eluent A: 50 mM sodium acetate buffer (pH 5.4) and eluent B: 100% acetonitrile. The injection volume was 1.0 µL, column temperature 30 °C, and the flow rate was 0.5 mL/min. For separation of AAS, a gradient was applied: Eluent B increased from 0 to 8% between 0 and 4.5 min followed by a cleaning procedure consisting of a rapid (10 s) increase to 80% eluent B, which were hold for 2 min, followed by a slow decline to 0% eluent B, which were hold for 1 min resulting in total run time of 12 min per sample. The concentration of AAS-ABA in the standard preparation was determined against a standard curve of ABA (0.05–1 µM). Assuming that the fluorescence emitted by 1 mol of ABA is equivalent to that emitted by 1 mol of derivatized protein carbonyl, a standard curve of AAS-ABA was prepared ranging from 0.1 to 1 µM. Regression coefficients greater than 0.999 were obtained for both the ABA and AAS-ABA standard curves. Identification and quantification of AAS in the MPI was carried out by comparing the retention times (Rt) in the FLD chromatograms with those from the standard AAS-ABA. The peak corresponding to AAS-ABA was manually integrated and the concentration of AAS in the MPI determined in pmol of carbonyl compound per mg MPI (*n* = 3).

### 2.10. Sensory Analysis

The brine-injected pork chops were evaluated in double sessions by a trained sensory panel consisting of 8 assessors, one male and 7 female, ranging between 4 to 21 years seniority. All the assessors had participated in two training session in accordance with ISO 4121, ASTM-MNL 13, DIN 13299 and were familiar with sensory assessment of meat. At the training sessions, the 14 descriptors were selected: pre-mature browning (PMB), hardness, tenderness, juiciness, off-flavor, stale flavor, warmed-over flavor, pig flavor, rancid, salt taste, sweet taste, sour flavor, acidic taste, meaty flavor. The descriptors were divided into two categories: *Appearance and texture*, and *Flavor*. All pork chops were analyzed on the day of sampling. Before cooking, the pork chops were tempered to 10–15 °C at room temperature and cooked on a pre-heated pan (170 °C) to a core temperature of 65–68 °C. Four pork chops were used per servings for the eight assessors, who evaluated the chops on a 15-point unstructured scale anchored at the extremes (0 = low intensity and 15 = high intensity). Twenty-four samples were evaluated per double session (4 brines × 6 replicates).

### 2.11. Statistical Data Analysis

Statistical analysis of salt content, ascorbate and phenol content, color, protein thiols, AAS, and protein cross-linking was performed using R^©^ version 3.4.2., The R Foundation for Statistical Computing, Vienna University of Economics and Business, Institute for Statistics and Mathematics, Vienna, Austria, ISBN: 3-900051-07-0. Analysis of variance were performed using a linear model with mixed effects with Brine, Time and Brine*Time as fixed effects, and Replicates as random effect, where brine, time and/or their interaction were found to be insignificant for the statistical model, and it was excluded as a variable. The level of significance was *p* < 0.05. Sensory data were analyzed using mixed models (SAS, 9.4). The proc mixed model included Brine, Time and Brine*Time as fixed effect and loin end, assessor, and assessor interactions as random effects. Least squares (LSmeans) were calculated and separated using probability of difference.

## 3. Results

### 3.1. Product Analyses

The salt content was analyzed in the pork chops prior to storage. In average, the salt concentration was 0.89 ± 0.15 g/100 g brine-injected meat with no significant difference between the different treatments (Table 1). In contrast, significant difference was found between samples prepared for the various storage days (Time, *p* = 0.0307), but this difference originated solely from a significant difference (*p* = 0.0155) between the control pork chops prepared for day 0 and day 7, containing 0.77 ± 0.06 g/100 g and 0.92 ± 0.11 g/100 g, respectively. The ascorbate and phenol concentrations in the brine-injected pork chops were calculated based on the weight gain after injection. The green tea extract had a total phenolic content of 23.8 g GAE/100 g extract [19], and the maté extract was found to contain 21.7 g GAE/100 g extract. The average concentration of salt, ascorbate and phenols, as well as the *p*-values for Time and Treatment are presented in Table 1.

No significant difference was found for ascorbate content between the three storage days, and for the phenolic content, no significant difference was found between the concentrations of green tea or maté extract, or between storage days (Table 1). This indicates a consistent production of brine-injected pork chops, where differentiation of the injection settings according to the hip- or neck-end of the loin, provided a homogenous application of ingredients.

### 3.2. Color Changes

The color of the brine-injected pork chops stored in high-oxygen MAP was found to differentiate significantly depending on storage time for the three-color parameters lightness (L*), redness (a*), and yellowness (b*) (Table 2).

The brine had no significant influence on the color parameters, and no significant interaction was found between brine and storage time. For all types of brines, the lightness was found to increase over time, leading to significant differences between day 0 and 3, but not between days 3 and 7, indicating that the main increase in lightness occurred during the first days of storage in high-oxygen MAP. Only pork chops added to green tea showed significant increase in lightness between days 3 and 7. Redness (a*) was found to be highest for all samples at day 3 followed by a significant decrease until day 7 for all samples because of discoloration, which may be assigned to oxidation of oxymyoglobin to metmyoglobin. In the pork loins added ascorbate, green tea or maté extracts significantly lower redness was observed at day 0 compared to day 3, which may be explained to increased blooming caused by high oxygen atmosphere (80%) at day 3 compared to atmospheric oxygen level (20%) at day 0. Yellowness (b*) was found to decrease significantly between day 0 and day 7 for the control pork loins. In contrast, for the pork loins added ascorbate or extracts, the highest value for yellowness were found at day 3 with a significant loss at day 7. The total color difference (ΔE) is a metric for understanding how the human eye perceives color difference, and values ~2.3 corresponds to JND (*just noticeable difference*) and values < 2.3 means that differences are not noticeable [21]. For the control and the pork chops injected with ascorbate or green tea ΔE increased over time, showing that storage in high-oxygen MAP affects perception of color in brine-injected pork. After three days of storage, all treatments resulted in JND, with the highest ΔE found for pork added mate (ΔE = 3.0). After 7 seven days of storage, the ΔE increased again for all treatments, except for maté, which dropped to 2.7.

### 3.3. Sensory Quality

The sensory quality of the brine-injected pork chops was evaluated by descriptors that can be divided into two categories: *Appearance and Texture*, and *Flavor*. The first category describes primarily quality parameters related to protein modifications (pigment and structural proteins), while the latter is related to the formation of secondary lipid oxidation products.

**Figure 1:**
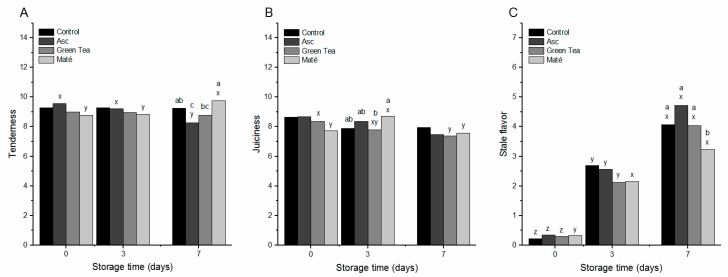
Tenderness (**A**); juiciness (**B**); and stale flavor (**C**) of pork chops injected with salt brine (Control), or brine added 225 ppm ascorbate (Asc), green tea (~25 ppm gallic acid equivalents (GAE)), or maté extract (~25 ppm GAE) and chill stored in high-oxygen modified atmosphere packaging (MAP) for 0, 3 or 7 as evaluated by a trained sensory panel. LSMeans with different letters (a–c) differ significantly (*p <* 0.05) between injection brines within the same day of storage, and LSMeans with different letters (x–z) differ significantly (*p* < 0.05) between days of storage within the same injection brine.

Within the category, *Appearance and Texture*, the descriptor tenderness was significantly affected by the interaction between storage time and brine, where the pork chops injected with ascorbate or maté extract showed opposite effects. The tenderness in the pork chops injected with ascorbate became significantly reduced between days 3 and 7, whereas the tenderness in the pork chops injected with maté extract increased significantly between days 3 and 7. This resulted in significant differences between samples at day 7, where pork chops injected with maté extract were more tender than the pork chops added ascorbate or green tea, and the pork chops added ascorbate was less tender than the control (Figure 1A).

The descriptor juiciness was also significantly affected by the interaction between storage time and brine. The pork chops injected with green tea extract was found to lose juiciness between days 3 and 7, whereas the pork chops injected with maté extract showed a peak in juiciness at day 3, followed by a drop at day 7 (Figure 1B). This resulted in significantly higher juiciness in the pork chops injected with maté extract as compared to the pork chops injected with green tea extract at day 3. Overall, the effects on tenderness and juiciness indicate that ascorbate, green tea and maté extracts all affect the texture and water holding capacity of the meat by interacting with the structural proteins, altering their functional properties, and, in the end, affects the eating quality of the meat. In particular, maté extract had a positive influence on meat tenderness and juiciness, whereas ascorbate had a negative influence on tenderness.

Concerning appearance of the meat, the descriptor pre-mature browning (PMB) was found to increase significantly during the seven days of storage in high-oxygen MAP (data not shown), as previously described by Soerheim and Hoey [30]. Furthermore, PMB was significantly higher for the pork injected with maté extract at day 0, indicating that the natural color of the maté extract (green), or some pro-oxidative interaction between maté extract and the pigments myoglobin, may have affected the appearance of the meat after cooking. Otherwise, no significant differences were observed between storage days or injection-brine, nor their interaction.

Within the category, *Flavor*, the descriptor stale flavor was also affected by the interaction between the injection-brine and storage time, and increased for the control pork chops and the pork chops injected with ascorbate or green tea extract during the seven days of storage (Figure 1). The pork chops injected with maté extract showed no significant increase from day 3 to 7, resulting in a significant reduced stale flavor at day 7 as compared to the three other treatments (Figure 1C). This indicated that the maté extract was able to retard the formation of stale flavor, which is considered a lipid oxidation derived off-flavor. Regarding the remaining descriptors within the category *Flavor*, they were all solely affected by storage time. Warmed-over-flavor, rancidity, and sour taste increased significantly during the seven days of storage in high-oxygen MAP, whereas acidity and meaty flavor decreased significantly (data not shown). Previous studies also show accelerated deterioration related to formation of lipid oxidation products caused by storage in the high-oxygen atmosphere [31,32]. In the present study, neither ascorbate, green tea, nor maté extracts protected against any of the lipid oxidation related descriptors besides stale flavor, which was significantly reduced by the maté extract. The only attribute found to be directly affected by injection brine was the attribute off-flavor, which was found to be significantly higher in the pork chops injected with green tea extract due to the flavor naturally found the green tea extract (data not shown).

From the sensory analysis, it is seen that descriptors such as acidity and meaty flavor are reduced already between day 0 and 3, where also warmed-over-flavor increases. Between days 3 and 7, changes are observed for sour taste and rancidity, which increases significantly, and the meat tenderness, which is either negatively affected by ascorbate or positively affected by maté extract.

### 3.4. Oxidative Protein Modifications

The protein thiol concentration in the myofibrillar protein isolate from the brine-injected pork chops was found to be similar for all treatments at day 0 (Table 3). No significant loss in thiols was observed after seven days of storage for the pork chops injected with green tea or maté extract. In contrast, ascorbate was found to significantly reduce the thiol concentration at days 3 and 7 as compared to day 0, indicating that ascorbate is unable to preserve protein thiols during storage. The semialdehyde AAS, which is a protein oxidation product of lysine, were also quantified in the brine-injected pork chops, and it was found that at day 0 the pork chops that added maté had a significantly higher concentration of AAS as compared to the other treatments, which showed similar concentrations (Table 3). No clear explanation was found for this difference at day 0 for the pork chops with added maté extract, and the level dropped again at day 3, where no significant differences were found between any of the treatments. After seven days of storage, the pork chops with added ascorbate, green tea and maté extract had all reached AAS concentrations significantly higher than the control, indicating prooxidative effects of both ascorbate and the extracts as compared to the control.

**Figure 2:**
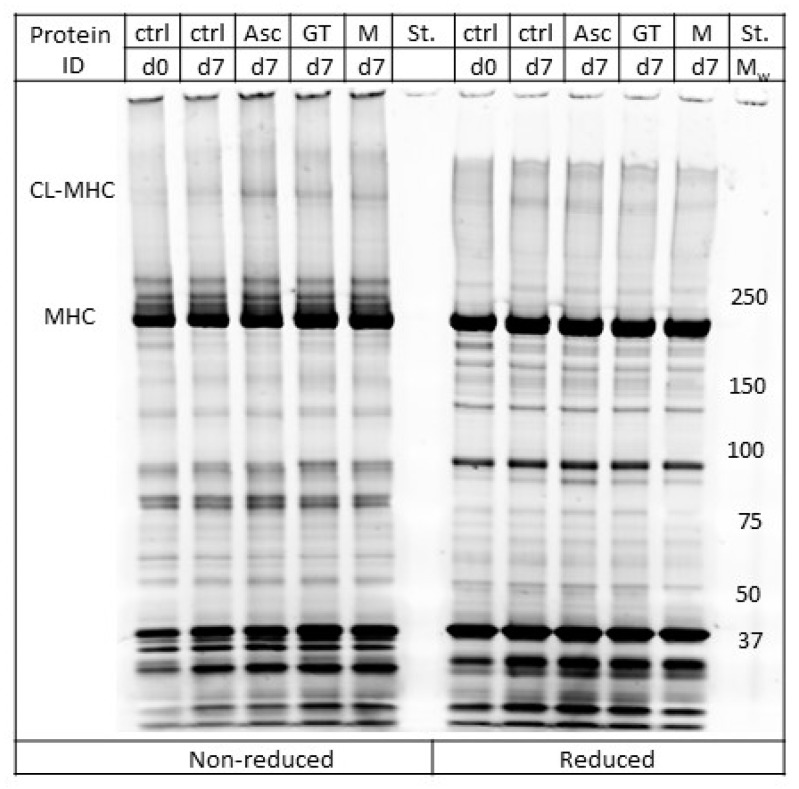
Representative SDS-PAGE of pork loins injected with neutral brine (ctrl) and chill stored in high-oxygen modified atmosphere packaging (MAP) for 0 (d0) or 7 days (d7), and pork loins injected with brine added 225 ppm ascorbate (Asc), green tea extract (GT; ~25 ppm gallic acid equivalents (GAE)), or maté extract (M; ~25 ppm GAE), and chill stored in high-oxygen (MAP) for seven days (d7). Samples were run both in their non-reduced state and reduced state. CL-MHC, cross-linked myosin heavy chain; MHC, myosin heavy chain.

The myofibrillar protein isolates from the brine-injected pork chops were also subjected to gel electrophoresis (SDS-PAGE) to evaluate the formation of protein cross-linking. Lund et al. [13] found that under oxidative conditions, myosin heavy chain (MHC) forms disulfide bonds, which cross-link the myosin heavy chains. The occurrence of cross-linked MHC (CL-MHC) was found to correlate with reduced tenderness and juiciness, and increased hardness in high-oxygen MAP pork *longissimus dorsi* [13]. Running the gel electrophoresis with samples in both their reduced and non-reduced state enabled the detection of disulfide-derived CL-MHC, as disulfides bonds will be reduced by the reducing agent thereby removing the protein cross-links and regenerating MHC. In the present study, control samples from day 0 and day 7 together with samples containing ascorbate, green tea, or maté extract from day 7 were analyzed by SDS-PAGE (Figure 2). For the non-reduced samples, a band assigned as the CL-MHC based on previous LC-MS analysis by Lund et al. [13] was detected for the pork loins added ascorbate, and to a lesser extent for the pork loins added green tea and maté extract at day 7. In contrast, almost no CL-MHC was visible in the reduced samples, indicating that the main part of the protein cross-links was generated through reducible bonds.

**Figure 3:**
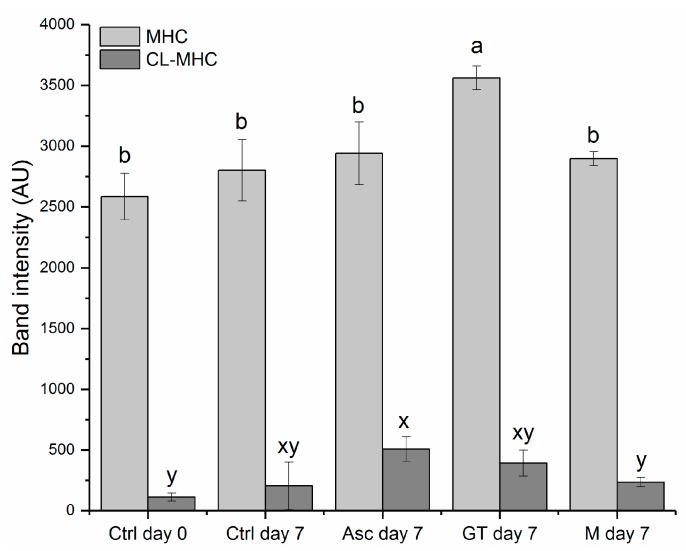
Band intensity of myosin heavy chain (MHC) and cross-linked MHC (CL-MHC) separated by SDS-PAGE in pork chops injected with neutral brine (Ctrl) and chill stored in high-oxygen modified atmosphere packaging (MAP) for 0 or 7 days, and pork loins injected with brine added 225 ppm ascorbate (Asc), green tea (GT, ~25 ppm gallic acid equivalents (GAE)), or maté extract (M, ~25 ppm GAE), and chill stored in high-oxygen (MAP) for seven days. Band intensity expressed as mean ± sd of three independent replicates. Means with different letters (a–b for MHC, and x–y for CL-MHC) differ significantly (*p <* 0.05).

Gel chromatography is not a quantitative method, and it is hence difficult to evaluate the exact difference between samples. This uncertainty is also due to the large variation between gels. In order to compare samples, the band intensities were quantified to obtain a semi-quantitative determination of MHC and CL-MHC. Addition of green tea extract to the brine-injected pork loins was found to significantly increase the MHC in the non-reduced samples at day 7 as compared to all the other samples (Figure 3). The same amount of protein was added to each well of the gel, so no clear explanation to this increment was found. Moreover, the green tea extract tended to increase the formation of CL-MHC, although the increase was not significantly different from the control (Figure 3). In contrast, ascorbate was found to significantly increase CL-MHC at day 7 as compared to the control sample at day 0, and the maté sample at day 7 (Figure 3). No significant differences in the CL-MHC levels were found between any samples after reduction (data not shown), indicating that the variations observed for CL-MHC between samples were caused by reducible bonds.

Overall, the results indicated that especially ascorbate, and to some extent green tea extract, increased the protein cross-links in the brine-injected pork chops during storage. However, as the gel chromatography results are merely semi-quantitative, they are less conclusive. Though, in this case, the results correlated well with the protein thiol concentrations determined and the tenderness evaluated by the sensory panel. Significantly lower thiol concentration was found at day 7 in the pork chops added ascorbate as compared to the other three pork chops on the same day (Table 3), confirming that thiols were involved in the protein cross-linking observed for the pork loins injected with ascorbate. Neither green tea nor maté extract were found to affect thiol loss or protein cross-linking significantly over seven days of storage, indicating no such prooxidative effect at the concentration level applied. It is known that oxidation of the phenolic moiety, *o*-catechols, results in the formation of electrophilic *o*-quinones that react rapidly with nucleophiles, such as protein thiol groups in meat proteins and generate protein-phenol adducts [33,34], and a similar reaction is also known for ascorbate with proteins generating a protein-DHA* adduct. After oxidation of ascorbate, dehydroascorbate (DHA) may readily decompose to a reactive 5-carbon compound DHA* that can modify reduced cysteinyl residues in proteins [35].

The reaction of quinones with meat proteins seems to be dose-dependent, as the loss of thiols depends on the concentration of phenolic compounds [36,37,38,39]. Preliminary studies in our groups found that the concentration level of 25 ppm phenols, which was used in the present study, minimized protein–quinone interactions as evident by no loss in protein thiols [17]. The low phenol concentration applied in the present study resulted in low to insignificant protection against lipid oxidation off-flavors as evaluated by the sensory panel. Only maté extract was able to protect significantly against stale flavor as compared to the control.

Green tea extract contains mainly catechin and catechin derivatives, while maté extracts contains chlorogenic acid and caffeic acid derivatives [14,15,16]. All compounds contain catechol moieties, which are excellent for protecting especially lipid against oxidation [14]. However, the catechol moeities may, under certain conditions, serve as prooxidants [40], as was seen for the generation of AAS in the present study by the addition of both extracts. Phenols from plant extracts are otherwise efficient radical scavengers, which protect lipids against oxidation in the water–lipid interfaces [41], but the results of the present study shows that only maté extract at the applied concentration served as potential antioxidant in brine-injected pork. However, it must be taken into consideration that components in the plant extracts may mask lipid oxidation derived flavors and therefore create a false antioxidant protection. In the present study, addition of green tea resulted in significant off-flavor formation related to the natural taste of the extract.

### 3.5. Ascorbate in Brine-Injected Pork

The results of the present study emphasized the link between MHC cross-linking in pork and product tenderness, as previously demonstrated for pork, beef, and lamb [13,42,43]. However, to our knowledge, this is the first demonstration of a clear correlation between protein cross-linking and tenderness in brine-injected pork chops. Addition of ascorbate to the injection brine promoted protein oxidation by increasing both AAS and thiol oxidation (Table 3), which led to increased protein cross-linking (Figure 3). Overall, these protein modifications resulted in reduced tenderness (Figure 1) directly by the formation of disulfide bonds due to thiol oxidation, and possibly also due to altered structural properties of the meat caused by other protein modifications, such as AAS [44]. In light of these results, ascorbate plays in this sense a double role in the brine-injected pork loins, on one hand added as an antioxidant to protect color and lipids, and on the other hand promoting protein oxidation as shown in the present study, and previously described for beef patties [45], and emulsion-type sausages [46]. This is a well-known phenomenon previously described on a cellular level [47]. In the aqueous phase, ascorbate may as an electron donor together with ascorbate oxidase activate oxygen to generate reactive oxygen species (ROS) and dehydroascorbate (DHA), which in proximity to proteins may oxidize thiols to generate disulfides or form protein-DHA* adducts [35]. Meanwhile, ascorbate is also known to reduce tocopheryl radicals to generate tocopherol and thereby act as an antioxidant protecting lipids from oxidation [47]. In the present study, the exact mechanism by which ascorbate results in thiol loss is unclear; however, the increased protein cross-linking observed indicates that ascorbate served as a prooxidant leading to protein cross-links rather than forming protein-DHA* adducts.

## 4. Conclusions

Substituting ascorbate in the production of brine-injected pork chops with phenolic-rich green tea or maté extracts showed equal efficiency against most lipid oxidation derived off-flavors. With regards to reduction of stale flavor, the maté extracts showed to be even more effective than green tea extract and ascorbate. Furthermore, ascorbate was found to increase protein thiols and protein cross-linking, with a concomitant reduction in meat tenderness at day 7 as compared to the control. Based on the present results, maté extract is a potential substitution for ascorbate in the production of brine-injected pork over green tea extract, as maté extract did not affect protein cross-linking, tenderness, or juiciness negatively throughout storage. Compared to green tea, maté extract generated no off-flavor, and, hence, based on the findings, is a valuable alternative as an antioxidant in brine-injected meat.

## Figures and Tables

**Table 1:** Concentration of NaCl and calculated levels of ascorbate and phenols compounds in brine-injected pork chops.

Treatment ^a^	*n*	NaCl	Ascorbate ^b^	Phenols ^b^	*p*_Time_
		(g/100 g)	(ppm)	(ppm)	
Control	6	0.85 ± 0.11	-	-	0.0307 (*)
Ascorbate (~225 ppm)	18	0.90 ± 0.18	225.6 ± 40.0	-	0.9878
Green Tea extract (~25 ppm GAE ^c^)	18	0.86 ± 0.12	-	24.0 ± 2.2	0.1260
Maté extract (~25 ppm GAE ^c^)	18	0.93 ± 0.15	-	24.5 ± 3.4	0.4670
*p*_Treatment_		0.3909	-	0.6141	

^a^ Stored in high-oxygen modified atmosphere packaging (MAP) for up to 7 days (5 °C); ^b^ Calculated from the weight-gain of brine-injected pork loins; ^c^ GAE = gallic acid equivalents; * Significant difference (*p <* 0.05).

**Table 2:** Color parameters and the total color difference (ΔE) in brine-injected pork chops.

Treatment ^x^	Storage Time	Lightness	Redness	Yellowness	ΔE
	(days)	(L*)	(a*)	(b*)	
Control (*n* = 6)	0	60.9 ± 1.5 ^b^	17.6 ± 0.6 ^a^	15.3 ± 0.2 ^a^	0.0
	3	63.2 ± 1.2 ^a^	18.0 ± 1.1 ^a^	14.9 ± 0.6 ^a^	2.4
	7	63.8 ± 1.5 ^a^	15.4 ± 1.1 ^b^	14.3 ± 0.6 ^b^	3.8
Ascorbate (~225 ppm, *n* = 6)	0	61.1 ± 2.0 ^b^	17.0 ± 0.6 ^b^	14.9 ± 0.3 ^a^	0.0
	3	63.4 ± 0.6 ^a^	18.2 ± 0.7 ^a^	15.3 ± 0.5 ^a^	2.6
	7	64.1 ± 1.3 ^a^	16.4 ± 1.0 ^b^	14.3 ± 0.5 ^b^	3.1
Green Tea extract (~25 ppm GAE ^y^, *n* = 6)	0	60.3 ± 1.4 ^c^	16.5 ± 0.8 ^b^	14.7 ± 0.7 ^a,b^	0.0
	3	62.8 ± 1.6 ^b^	17.8 ± 1.1 ^a^	15.2 ± 0.5 ^a^	2.8
	7	64.0 ± 1.0 ^a^	15.4 ± 0.9 ^c^	14.3 ± 0.5 ^b^	3.8
Maté extract (~25 ppm GAE ^y^, *n* = 6)	0	60.5 ± 1.2 ^b^	16.7 ± 0.5 ^b^	14.6 ± 0.2 ^a,b^	0.0
	3	62.9 ± 1.1 ^a^	17.9 ± 0.9 ^a^	14.9 ± 0.6 ^a^	3.0
	7	62.9 ± 1.3 ^a^	15.8 ± 0.8 ^b^	14.1 ± 0.4 ^b^	2.7

^x^ Stored in high-oxygen modified atmosphere packaging (MAP) for up to 7 days (5 °C); ^y^ GAE = gallic acid equivalents; ^a–c^ Different letters (a–c) denotes significant (*p* < 0.05) different values within the same treatment and color parameter.

**Table 3:** Protein thiols and α-amino-adipic semialdehyde (AAS) in myofibrillar protein isolate (MPI) from brine-injected pork.

Marker of Protein Oxidation	Storage Time *	Control	Ascorbate	Green Tea Extract	Maté Extract
	(days)		(~225 ppm)	(~25 ppm GAE **)	(~25 ppm GAE **)
Protein thiols (nmol/mg protein, *n* = 6)	0	62.2 ± 2.2	61.2 ± 1.8 ^x^	61.5 ± 1.6 ^x,y^	63.9 ± 2.1 ^x^
	3	59.4 ± 3.6 ^a^	56.6 ± 3.4 ^b,y^	59.0 ± 2.6 ^a,y^	58.1 ± 2.8 ^a,b,y^
	7	60.6 ± 1.7 ^a^	57.8 ± 1.7 ^b,y^	61.7 ± 1.7 ^a,x^	61.0 ± 0.7 ^a,y,x^
AAS (pmol/mg MPI, *n* = 3)	0	62.5 ± 0.0 ^b^	61.1 ± 7.3 ^b,x^	55.4 ± 12.4 ^b,y^	112.5 ± 10.2 ^a,x^
	3	82.2 ± 15.3	108.9 ± 17.5 ^x^	104.5 ±3.1 ^x^	80.5 ± 3.0 ^y^
	7	64.2 ± 4.6 ^b^	114.2 ± 27.5 ^a,x^	110.9 ± 20.6 ^a,x^	129.2 ± 9.4 ^a,x^

* Stored in high-oxygen modified atmosphere packaging (MAP) for up to 7 days (5 °C); ** GAE = gallic acid equivalents. ^a–b^ Mean ± sd within the same row without a common letter differ significantly (*p <* 0.05). ^x–y^ Mean ± sd within the same analysis and within the same column without a common letter differ significantly (*p <* 0.05).

## References

[1] Sheard P.R., Tali A. (2004). Injection of salt, tripolyphosphate and bicarbonate marinade solutions to improve the yield and tenderness of cooked pork loin. Meat Sci..

[2] Walsh H., Martins S., O’Neill E.E., Kerry J.P., Kenny T., Ward P. (2010). The effects of different cooking regimes on the cook yield and tenderness of non-injected and injection enhanced forequarter beef muscles. Meat Sci..

[3] Grobbel J.P., Dikeman M.E., Hunt M.C., Milliken G.A. (2008). Effects of different packaging atmospheres and injection-enhancement on beef tenderness, sensory attributes, desmin degradation, and display color. J. Anim. Sci..

[4] Suman S.P., Hunt M.C., Nair M.N., Rentfrow G. (2014). Improving beef color stability: Practical strategies and underlying mechanisms. Meat Sci..

[5] Cheng E.H., Ockerman H.W. (2004). Effect of ascorbic acid with tumbling on lipid oxidation of precooked roast beef 1. J. Muscle Food.

[6] Sato K., Hegarty G.R. (1971). Warmed-over flavor in cooked meats. J. Food Sci..

[7] Mitsumoto M., O’Grad M.N., Kerry J.P., Buckley D.J. (2005). Addition of tea catechins and vitamin C on sensory evaluation, colour and lipid stability during chilled storage in cooked or raw beef and chicken patties. Meat Sci..

[8] Racanicci A.M.C., Menten J.F.M., Alencar S.M., Buissa R.S., Skibsted L.H. (2011). Mate (*Ilex paraguariensis*) as dietary additive for broilers: Performance and oxidative stability of meat. Eur. Food Res. Technol..

[9] Gravador R.S., Jongberg S., Andersen M.L., Luciano G., Priolo A., Lund M.N. (2014). Dietary citrus pulp improves protein stability in lamb meat stored under aerobic conditions. Meat Sci..

[10] Jongberg S., Skov S.H., Tørngren M.A., Skibsted L.H., Lund M.N. (2011). Effect of white grape extract and modified atmosphere packaging on lipid and protein oxidation in chill stored beef patties. Food Chem..

[11] Mielnik M.B., Sem S., Egelandsdal B., Skrede G. (2008). By-products from herbs essential oil production as ingredient in marinade for turkey thighs. LWT Food Sci. Technol..

[12] Kim Y.J., Jin S.K., Park W.Y., Kim B.W., Joo S.T., Yang H.S. (2010). The effect of garlic or onion marinade on the lipid oxidation and meat quality of pork during cold storage. J. Food Qual..

[13] Lund M.N., Lametsch R., Hviid M.S., Jensen O.N., Skibsted L.H. (2007). High-oxygen packaging atmosphere influences protein oxidation and tenderness of porcine *longissimus dorsi* during chill storage. Meat Sci..

[14] Brewer M.S. (2011). Natural Antioxidants: Sources, Compounds, Mechanisms of Action, and Potential Applications. Compr. Rev. Food Sci. Food Saf..

[15] Heck C.I., De Mejia E.G. (2007). Yerba Mate tea (*Ilex paraguariensis*): A comprehensive review on chemistry, health implications, and technological considerations. J. Food Sci..

[16] De Zawadzki A., Arrivetti L.O.R., Vidal M.P., Catai J.R., Nassu R.T., Tullio R.R., Berndt A., Oliveira C.R., Ferreira A.G., Neves-Junior L.F. (2017). Mate extract as feed additive for improvement of beef quality. Food Res. Int..

[17] Jongberg S., Mari Ann T., Skibsted L.H. (2018). Dose-Dependent Effects of Green Tea or Maté Extracts on Lipid and Protein Oxidation in Brine-Injected Retail-Packed Pork Chops. Medicines.

[18] Singleton V.L., Rossi J.A. (1965). Colorimetry of total phenolics with phosphomolybdic-phosphotungstic acid reagents. Am. J. Enol. Vitic..

[19] Jongberg S., Lund M.N., Østdal H., Skibsted L.H. (2012). Phenolic Antioxidant Scavenging of Myosin Radicals Generated by Hypervalent Myoglobin. J. Agric. Food Chem..

[20] Nordisk Metodikkomite for Levnedsmidler Chloride (Salt) (2004). Determination in Foods by Potentiometric Titration.

[21] Sharma G., Bala R. (2002). Digital Color Imagine Handbook.

[22] Park D., Xiong Y.L.L., Alderton A.L. (2007). Concentration effects of hydroxyl radical oxidizing systems on biochemical properties of porcine muscle myofibrillar protein. Food Chem..

[23] Koutina G., Jongberg S., Skibsted L.H. (2012). Protein and lipid oxidation in Parma ham during production. J. Agric. Food Chem..

[24] Ellman G.L. (1959). Tissue sulfhydryl groups. Arch. Biochem. Biophys..

[25] Jongberg S., Wen J., Tørngren M.A., Lund M.N. (2014). Effect of high-oxygen atmosphere packaging on oxidative stability and sensory quality of two chicken muscles during chill storage. Food Packag. Shelf Life.

[26] Akagawa M., Sasaki D., Ishii Y., Kurota Y., Yotsu-Yamashita M., Uchida K., Suyama K. (2006). New method for the quantitative determination of major protein carbonyls, alpha-aminoadipic and gamma-glutamic semialdehydes: Investigation of the formation mechanism and chemical nature in vitro and in vivo. Chem. Res. Toxicol..

[27] Akagawa M., Suyama K., Uchida K. (2009). Fluorescent detection of a-aminoadipic and g-glutamic semialdehydes in oxidized proteins. Free Radic. Biol. Med..

[28] Estévez M., Morcuende D., Ventanas S. (2009). Determination of oxidation. Handbook of Processed Meats and Poultry Analysis.

[29] Utrera M., Morcuende D., Rodríguez-Carpena J.G., Estévez M. (2011). Fluorescent HPLC for the detection of specific protein oxidation carbonyls-α-aminoadipic and γ-glutamic semialdehydes—In meat systems. Meat Sci..

[30] Soerheim O., Hoey M. (2013). Effects of food ingredients and oxygen exposure on premature browning in cooked beef. Meat Sci..

[31] Clausen I., Jakobsen M., Ertbjerg P., Madsen N.T. (2009). Modified atmosphere packaging affects lipid oxidation, myofibrillar fragmentation index and eating quality of beef. Packag. Technol. Sci..

[32] Zakrys P.I., Hogan S.A., O’Sullivan M.G., Allen P., Kerry J.P. (2008). Effects of oxygen concentration on the sensory evaluation and quality indicators of beef muscle packed under modified atmosphere. Meat Sci..

[33] Rawel H.M., Kroll J., Hohl U.C. (2001). Model studies on reactions of plant phenols with whey proteins. Mol. Nutr. Food Res..

[34] Jongberg S., Lund M.N., Waterhouse A.L., Skibsted L.H. (2011). 4-Methyl catechol inhibits protein oxidation in meat but not disulfide formation. J. Agric. Food Chem..

[35] Kay P., Wagner J.R., Gagnon H., Day R., Klarskov K. (2013). Modification of Peptide and Protein Cysteine Thiol Groups by Conjugation with a Degradation Product of Ascorbate. Chem. Res. Toxicol..

[36] Cao Y., Xiong Y.L. (2015). Chlorogenic acid-mediated gel formation of oxidatively stressed myofibrillar protein. Food Chem..

[37] Feng X., Chen L., Lei N., Wang S., Xu X., Zhou G., Li Z. (2017). Emulsifying Properties of Oxidatively Stressed Myofibrillar Protein Emulsion Gels Prepared with (−)-Epigallocatechin-3-gallate and NaCl. J. Agric. Food Chem..

[38] Jia N., Wang L., Shao J., Liu D., Kong B. (2017). Changes in the structural and gel properties of pork myofibrillar protein induced by catechin modification. Meat Sci..

[39] Jongberg S., Terkelsen L.D., Miklos R., Lund M.N. (2015). Green tea extract impairs meat emulsion properties by disturbing protein disulfide cross-linking. Meat Sci..

[40] Zhou L., Elias R.J. (2011). Investigating the hydrogen peroxide quenching capacity of proteins in polyphenol-rich foods. J. Agric. Food Chem..

[41] Shah M.A., Bosco S.J.D., Mir S.A. (2014). Plant extracts as natural antioxidants in meat and meat products. Meat Sci.

[42] Zakrys-Waliwander P.I., O’Sullivan M.G., O’Neill E.E., Kerry J.P. (2012). The effects of high oxygen modified atmosphere packaging on protein oxidation of bovine, *M. longissimus dorsi* muscle during chilled storage. Food Chem..

[43] Kim Y.H., Huff-Lonergan E., Sebranek J.G., Lonergan S.M. (2010). High-oxygen modified atmosphere packaging system induces lipid and myoglobin oxidation and protein polymerization. Meat Sci..

[44] Estévez M. (2011). Protein carbonyls in meat systems: A review. Meat Sci..

[45] Lund M.N., Hviid M.S., Skibsted L.H. (2007). The combined effect of antioxidants and modified atmosphere packaging on protein and lipid oxidation in beef patties during chill storage. Meat Sci..

[46] Rysman T., Van Hecke T., De Smet S., Van Royen G. (2016). Ascorbate and Apple Phenolics Affect Protein Oxidation in Emulsion-Type Sausages during Storage and in Vitro Digestion. J. Agric. Food Chem..

[47] Bánhegyi G., Csala M., Szarka A., Varsányi M. (2003). Role of ascorbate in oxidative folding. Biofactors.

